# Assay of Endogenous 3,5-diiodo-L-thyronine (3,5-T_2_) and 3,3′-diiodo-L-thyronine (3,3′-T_2_) in Human Serum: A Feasibility Study

**DOI:** 10.3389/fendo.2019.00088

**Published:** 2019-02-19

**Authors:** Leonardo Lorenzini, Nhat Minh Nguyen, Ginevra Sacripanti, Enrico Serni, Marco Borsò, Federica Saponaro, Elena Cecchi, Tommaso Simoncini, Sandra Ghelardoni, Riccardo Zucchi, Alessandro Saba

**Affiliations:** ^1^Department of Pathology, University of Pisa, Pisa, Italy; ^2^Department of Clinical and Experimental Medicine, University of Pisa, Pisa, Italy

**Keywords:** thyroid hormones, thyroid hormones metabolites, 3, 5-diiodo-L-thyronine, mass spectrometry, T_2_

## Abstract

3,5-diiodo-L-thyronine (3,5-T2) is an endogenous derivative of thyroid hormone with potential metabolic effects. It has been detected in human blood by immunological methods, but a reliable assay based on mass spectrometry (MS), which is now regarded as the gold standard in clinical chemistry, is not available yet. Therefore, we aimed at developing a novel *ad-hoc* optimized method to quantitate 3,5-T2 and its isomers by MS in human serum. Serum samples were obtained from 28 healthy subjects. Two ml of serum were deproteinized with acetonitrile and then subjected to an optimized solid phase extraction-based procedure. To lower background noise, the samples were furtherly cleaned by hexane washing and acetonitrile precipitation of residual proteins. 3,5-T2 and its isomers 3,3′-T2 and 3′,5′-T2 were then analyzed by HPLC coupled to tandem MS. Accuracy and precision for T2 assay were 88–104% and 95–97%, respectively. Recovery and matrix effect averaged 78% and +8%, respectively. 3,5-T2 was detected in all samples and its concentration averaged (mean ± SEM) 41 ± 5 pg/ml, i.e., 78 ± 9 pmol/l. In the same samples the concentration of 3,3′-T2 averaged 133±15 pg/ml, i.e., 253±29 pmol/l, while 3′,5′-T2 was not detected. 3,5-T2 concentration was significantly related to 3,3′-T2 concentration (*r* = 0.540, *P* < 0.01), while no significant correlation was observed with either T3 or T4 in a subset of patients in which these hormones were assayed. In conclusion, our method is able to quantify 3,5-T2 and 3,3′-T2 in human serum. Their concentrations lie in the subnanomolar range, and a significant correlation was detected between these two metabolites in healthy individuals.

## Introduction

The term thyroid hormones, classically referred to both 3,5,3′-triiodothyronine (T_3_) and thyroxine (T_4_), seems nowadays to be simplistic; indeed, it has been shown that some T_3_ and T_4_ metabolites, particularly 3,5-diiodothyronine (3,5-T_2_) and 3-iodothyronamine, are independent chemical messengers, with specific metabolic effects (1).

Two deiodinase enzymes, namely D1- and D3-deiodinase, can potentially catalyse the synthesis of distinct diiodothyronines: 3,5-diiodothyronine (3,5-T_2_), 3,3′- diiodothyronine (3,3′-T_2_) and 3′,5′- diiodothyronine (3′,5′-T_2_) (2). Until recent years, all T_2_ isomers were regarded as inactive metabolites of T_3_ and T_4_, because of their very low affinity for nuclear thyroid hormone receptors. However, this view has been challenged by the observation that 3,5-T_2_ can also interact with mitochondrial targets and that administration of exogenous 3,5-T_2_ to experimental animals produces significant functional effects on lipid metabolism and mitochondrial function (3).

A major pitfall in the investigations about the alleged physiological and pathophysiological relevance of 3,5-T_2_ is the difficulty in assaying its low serum or tissue concentration, either in animal models or in human.

Polyclonal antibody-based radio-immunoassays were developed in the 1970's. The first radio-immunoassay from Meinhold et al. allowed detection of serum 3,5-T_2_ in 10 normal subject (100 pmol/L) and in 5 hyperthyroid patients (380 nmol/L) (4). Subsequently, in 1982 Faber et al. reported a serum 3,5-T_2_ concentration close to 100 pmol/L with a RIA method based on gel separation and antibody extraction (GSAE) (5). Further RIA methods have been subsequently employed, but high variability was reported for serum 3,5-T_2_ concentration, i.e., between 10 and 190 pmol/L (6). In 2014, Lehmphul et al. developed a new CLIA method for 3,5-T_2_ evaluation with a lower detection limit of 200 pmol/L. The average concentration was 430 pmol/L in 31 hypothyroid patients and 310 pmol/L in 24 hyperthyroid patients, vs. 290 pmol/L in the control healthy group, although this difference was not statistically significant (7).

Mass spectrometry-based methods should provide a more specific and sensitive quantification technology for the assay of thyroid hormone and their metabolites, including 3,5-T_2_. In recent years, several groups have published method for 3,5-T_2_ detection based on Liquid Chromatography-tandem Mass Spectrometry (LC-MS-MS) (8), but so far, the application of these techniques to human serum samples usually produced negative results.

Due to these methodological challenges, the aim of this study was to develop an offline pre-analytical sample preparation (sample extraction) procedure and an LC-MS-MS method for quantification of T_2_ isomers in human serum.

## Materials and Methods

### Sample Collection

Serum samples were obtained from 28 patients, including 8 healthy volunteers and 20 women undergoing endocrinological screening and found to be euthyroid. Serum was obtained by using the remaining part of samples obtained for independent clinical indications. The study had the approval of the local Ethical Committee. All subjects gave a written informed consent and since no additional blood drawings were performed, ethical committee approval was not required.

### Reagents and Materials

3,3′,5,5-tetraiodo-L-thyronine (T_4_), 3,3′,5-triiodo-L-thyronine (T_3_), 3,3′,5′-triiodo-L-thyronine (rT_3_), 3,5-diiodo-L-thyronine (3,5-T_2_), 3,3′-diiodo-L-thyronine (3,3′-T_2_), 3,3′,5,5-tetraiodo-L-thyronine-^13^C_6_ (^13^C_6_-T_4_), and 3,3′,5-triiodo-L-thyronine-^13^C_6_ (^13^C_6_-T_3_), were provided by Sigma-Aldrich (Saint Louis, MO, USA). Acetonitrile (LC-MS grade), methanol (LC-MS grade), ultra-pure water (LC-MS grade), hexane (HPLC grade), glacial acetic acid (ACS grade), hydrochloric acid (ACS grade), and ammonium hydroxide (ACS grade), were also from Sigma -Aldrich. 3,3′-diiodo-L-tyronine-^13^C_6_ (^13^C_6_-T_2_) was purchased from Isosciences (Ambler, PA, USA), while 3,5-diiodo-L-tyronine-^13^C_9_-^15^N (^13^${\text{C}}_{9}^{15}$N-T_2_) was kindly provided by Prof. Thomas S. Scanlan (Portland, OR, USA).

### Sample Preparation

The optimization of the sample preparation procedure, as well as the instrumental method development, took advantage of the expertise gained in the assay of T_4_, T_3_, rT_3_, and some of their metabolites in human and animal biological matrices (9–11). The general strategy of the pre-analytical procedure consisted in using a relatively large amount of starting material (human serum) that was concentrated up to 40-fold. Since this approach tends to generate a high matrix effect, additional steps were introduced to ensure extensive cleaning of the sample, both before and after the extraction step. Briefly, 2 mL of serum from each sample were placed in a 2 mL microcentrifuge tube (Eppendorf, Hamburg, Germany) and spiked with appropriate internal standards amount in order to achieve the final concentration of 1 ng/mL, 5 ng/mL, 50 ng/mL, and 200 ng/mL for ^13^C_6_-T_2_, ^13^${\text{C}}_{9}^{15}$N-T_2_, ^13^C_6_-T_3_, and ^13^C_6_-T_4_, respectively. After a 2 min shaking at room temperature, the sample was split into 4 aliquots of 500 μL each, and all of them was added with 500 μL of a mixture containing ice-cold acetonitrile (79% as v/v), water (20%), and formic acid (1%) for deproteinization. After vortexing and sonication (15 min), 1 mL of ice-cold acetonitrile was added to each aliquot, which was then shacked for 2 min, sonicated for 15 min, and centrifuged for 15 min at 14,000 × g. The resulting supernatants were warmed up to 40°C and evaporated up to ~0.5 mL under a gentle stream of nitrogen. The liquid residues from the different aliquots of the same sample were pooled and loaded onto an Agilent (Santa Clara, CA, USA) Bond-Elut Certify 130 mg SPE cartridge, previously conditioned by sequential wetting with 2 mL of methanol and 3 mL of water. After washing with 2 mL of water, 2 mL of 0.1 M HCl, and 5 mL of methanol, the column was dried, and the sample was eluted with 2 mL of methanol/ammonium hydroxide (95:5 by volume). The eluate was dried under a gentle stream of nitrogen and reconstituted with 200 μL of water/acetonitrile (70:30) containing 0.1% formic acid, and then shaken for 6 min at room temperature. The reconstituted solution was washed with 600 μL of hexane to extract lipid residues, which were then removed, while the aqueous solution containing the analytes was added with 500 μL acetonitrile, shaken for 2 min, and centrifuged for 15 min. After removing the pellet, the supernatant was dried under nitrogen and stored at −20^o^C until processing. Immediately before analysis, it was reconstituted with 50 μL of water/acetonitrile (70:30) containing 0.1% formic acid, shaken for 15 min, centrifuged for 15 min and injected (20 μL of the supernatant) into the HPLC-MS-MS system.

### Instrumental Layout and Operative Conditions

Samples were processed by using an instrument layout consisting of an AB Sciex API 4000 triple quadrupole mass spectrometer (Concord, ON, Canada), equipped with an electrospray (ESI) Turbo V ion source, coupled to an Agilent 1290 Infinity UHPLC system (Santa Clara, CA, USA), including autosampler outfitted with peltier tray, binary pump, and column oven, used for the chromatographic separation. A ten port divert valve (Valco Instruments Co. Inc., Huston, TX, USA) allowed the discarding of both head and tail of the HPLC runs, while a quaternary HPLC pump (Series 200, PerkinElmer, Boston, MA, USA) supplied the mass spectrometer with solvent when the flow from the analytical system was diverted to waste, to prevent the occurrence of high voltage discharge in the ion source. Chromatographic separation was performed by a Phenomenex (Torrance, CA, USA) Gemini C18 110 Å, 3 μm, 50 x 2 mm HPLC column protected by a C18 4 x 2.0 mm ID security guard cartridge. Data acquisition and system control were carried out by an AB Sciex Analyst^®^ version 1.6.3 software.

Twenty micro liters of each sample was injected into the Agilent UPLC system, where it was submitted to chromatographic separation by a mixture methanol/acetonitrile (20/80) added with 0.1% formic acid, as solvent A, and water also containing 0.1% of formic acid, as solvent B, under the gradient conditions shown in Table 1. The chromatographic column was kept at 20°C.

**Table 1:** Program of HPLC pumps.

	**HPLC Quaternary Pump**				**HPLC Binary Pump**			
**Step**	**Total time (min)**	**Flow rate (μl/min)**	**Methanol (%)**	**Water (%)**	**Total time (min)**	**Flow rate (μl/min)**	**Solvent A (%)**	**Solvent B (%)**
0	0.1	50	50	50	0.1	400	5	95
1	3.0	200	50	50	3.0	400	5	95
2	9.5	50	50	50	8.5	400	65	35
3	13.5	50	50	50	9.0	400	100	0
4					11.0	400	100	0
5					11.5	400	5	95
6					13.5	400	5	95

Mass spectrometry method was based on selected reaction monitoring (SRM) in positive ion mode and made use of three transitions for each compound, one of which was used as a quantifier (Q) and the others as qualifier (q). All of them were monitored using optimized declustering potential (DPs), collision energies (CEs), and collision exit potentials (CXPs), which are reported in Table 2. Further operative parameters were: nebulizer current (NC), 5.0 KV; gas source 1 (GS1) zero air, 70; gas source 2 (GS2) zero air, 55; source temperature (TEM), 650°C; entrance potential (EP), 10 V; IQ1 lens potential, −9.8 V; stubby rod Q1 (ST),−15.5 V; collision gas (CAD) nitrogen, operative pressure with CAD gas on, 5.7 mPa.

**Table 2:** Mass spectrometry operative parameters.

**Analyte**	**SRM transition**	**Operative Parameters**		
		**DP**	**CE**	**CXP**
3,5-T_2_; 3,3′-T_2_	525.9→ 352.9 (q)	87	40.8	10.3
	525.9→ 381.8 (q)		27.6	11.2
	525.9→ 479.9 (Q)		26.0	14.2
T_3_; rT_3_	651.8→ 478.9 (q)	76	47.7	13.7
	651.8→ 508.0 (q)		31.2	14.8
	651.8→ 605.9 (Q)		31.3	17.9
T_4_	777.8→ 604.8 (q)	82	52.8	17.0
	777.8→ 633.9 (q)		36.0	18.6
	777.8→ 731.9 (Q)		34.0	22.0
^13^C_6_-T_2_	531.9→ 358.9 (q)	87	40.8	10.3
	531.9→ 387.9 (q)		27.6	11.2
	531.9→ 485.9 (Q)		26.0	14.2
^13^${\text{C}}_{9}^{15}$N-T_2_	535.9→ 361.9 (q)	87	40.8	10.3
	535.9→ 390.8 (q)		27.6	11.2
	535.9→ 488.9 (Q)		26.0	14.2
^13^C_6_-T_3_	657.8→ 484.9 (q)	76	47.7	13.7
	657.8→ 514.0 (q)		31.2	14.8
	657.8→ 611.9 (Q)		31.3	17.9
^13^C_6_-T_4_	783.8→ 610.8 (q)	82	52.8	17.0
	783.8→ 639.9 (q)		36.0	18.6
	783.8→ 737.9 (Q)		34.0	22.0

### Method Performances

Quality control was performed according to Matuszewski et al. (12). Accuracy is the ratio of measured concentration to spiked concentration after adding known amounts of analytes at two different concentration levels: precision is the coefficient of variation (standard deviation/mean) of repeated measurements within the same assay under the same conditions as described above; recovery is defined as the ratio of internal standard spiked before extraction to internal standard spiked after extraction; matrix effect is defined as the ratio of internal standard spiked after extraction to internal standard dissolved in the reconstitution solvent.

Statistical analysis was performed with GraphPad Prism version 7.0 for Windows (GraphPad Software, San Diego, CA). Data are reported as mean ± SEM. Correlation analysis was performed through linear regression, taking *P* = 0.05 as the conventional limit of statistical significance.

## Results

Quality control data for T_2_ assay averaged as follows, without apparent differences between isomers: accuracy 88–104%, precision 95–97%, recovery 78%, matrix effect +8%. For T_3_ and T_4_, recovery and precision were 104–128 and 85–94%, respectively. Recovery, matrix effect and precision averaged 67, −15, and 115% for T_3_; 44, −7, and 90% for T_4_. The lower limit of detection, determined with a signal to noise ratio of 3, was 11.5 pg/mL (22 pmol/L) for T_2_, 9.1 pg/mL (14 pmol/L) for T_3_, and 8.5 pg/mL (11 pmol/L) for T_4_.

**Figure 1:**
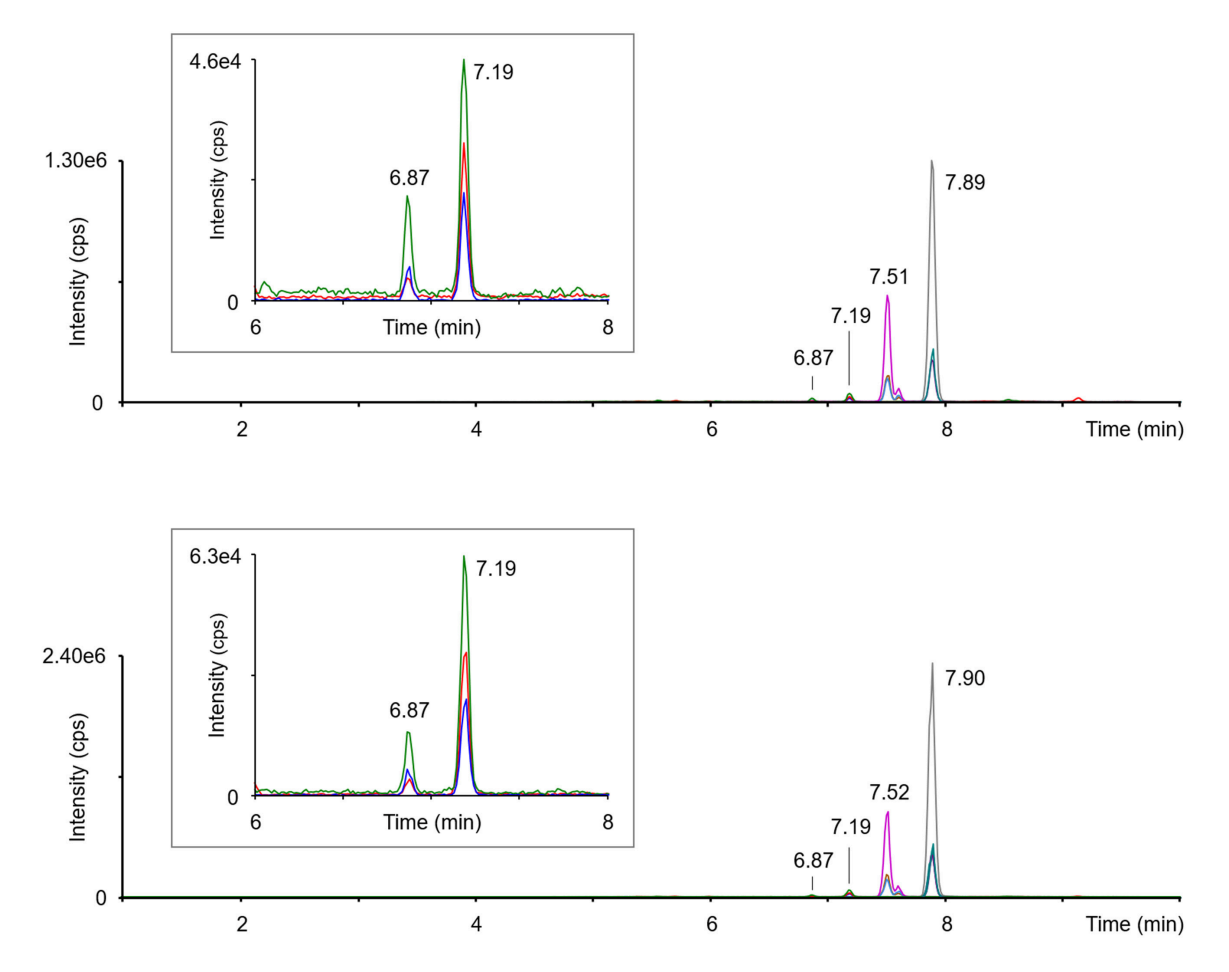
Representative chromatograms obtained in two different subjects. The green, red, and blue tracings, reported also as an expanded view in the framed panels, refer to the three transitions monitored for 3, 5-T_2_ (6.87min.) and 3,3′-T_2_ (7.19min.), namely *m/z* 529.9 → 352.9, 529.9 → 381.8, and 525.9 → 479.9. Three more peaks are attributable to T_3_ (7.51–7.52min.), rT_3_ (small peak next to T_3_, at 7.60–7.61min), and T4 (7.89–7.90min.). Peak identity was confirmed by the comparison to appropriate standards, as detailed in the methods section.

Representative chromatograms obtained in two patients are shown in Figure 1. 3,5-T_2_ and 3,3′-T_2_ were present in all samples, while 3′,5′-T_2_ was never detected. Peak identity was determined by comparison with appropriate standard based on retention times and ratio between the three transitions.

**Figure 2:**
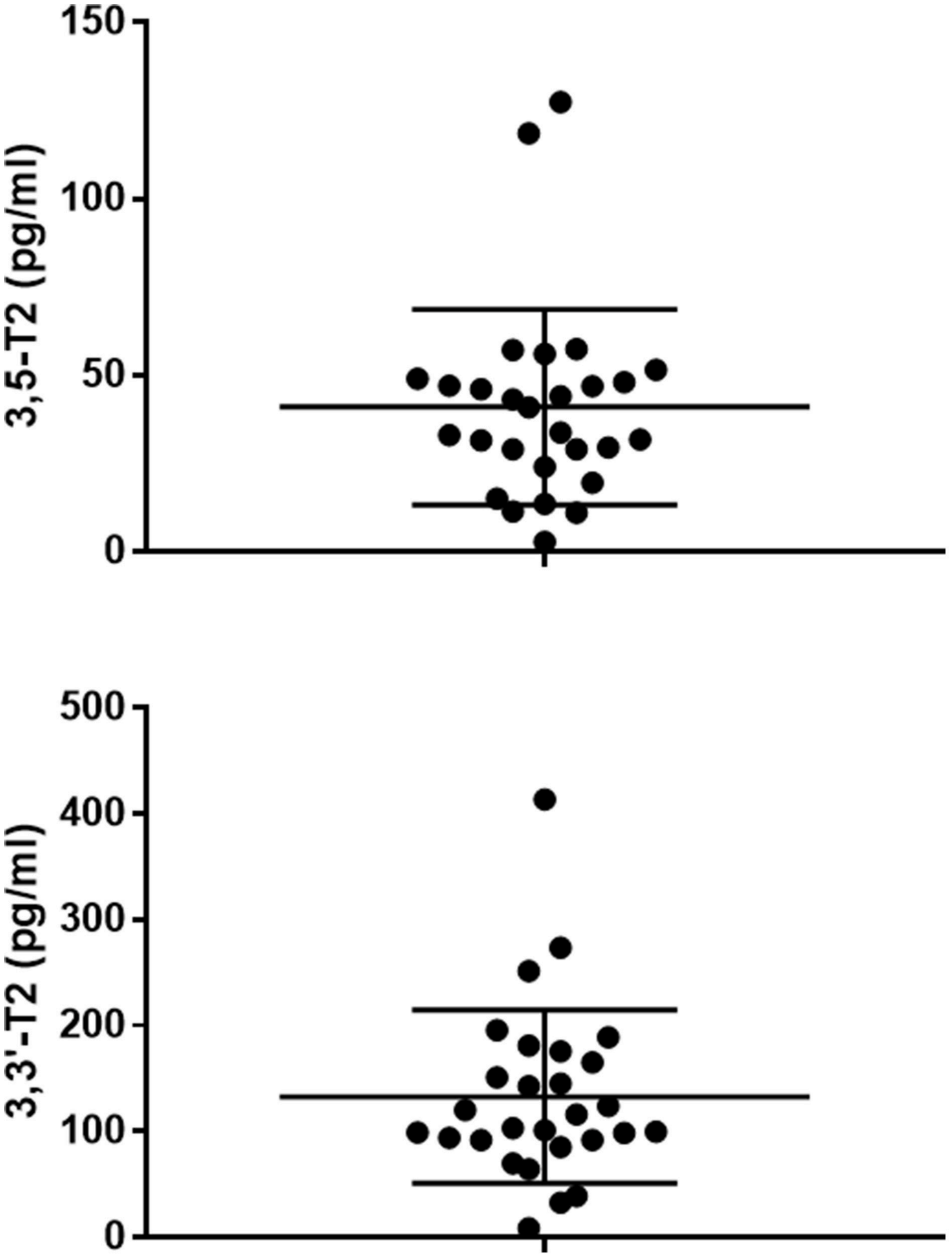
Scatter plots showing the serum concentrations of 3,5-T_2_ (upper panel) and 3,3′-T_2_ (lower panel) in the 28 patients included in our series. The horizontal lines represent mean ± SD. Please note a different scale was used in the two panels.

Analyte concentration was determined by appropriate calibration curves and was based on the first transition, namely Q transition in Table 2. Scatter plot of the results obtained for 3,5-T_2_ and 3,3′-T_2_ are shown in Figure 2. 3,5-T_2_ concentration averaged 41±5 pg/mL, i.e., 78 ± 9 pmol/L. In the same samples the concentration of 3,3′-T_2_ was about 3-fold higher and averaged 133±15 pg/mL, i.e., 253±29 pmol/L.

**Figure 3:**
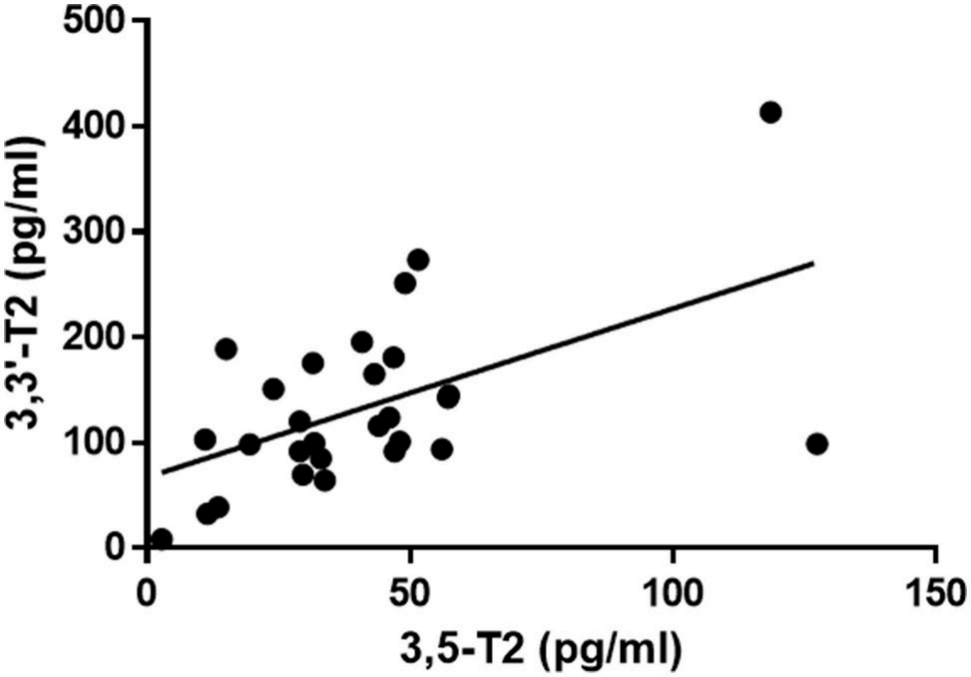
Scatter plot of the relationship between 3,5-T_2_ and 3,3′-T_2_ serum concentration in the 28 patients included in our series. Linear regression analysis yielded *r* = 0.540 with *P* = 0.003. The regression line is plotted (y = ax + b, with a = 1.600 ± 0.489 and b = 67.29 ± 24.06 pg/mL).

As shown in Figure 3, 3,5-T_2_ concentration was significantly related to 3,3′-T_2_ concentration (*r* = 0.540, *P* < 0.01), while no significant correlation was observed with either T_3_ or T_4_ in the subset of patients in which these hormones were assayed.

## Discussion

Due to the emerging role of 3,5-T_2_ in lipid and glucose metabolism, the adequate measurement of 3,5-T_2_ and related compound is a challenging aim of thyroid hormone research. This is necessary to identify the clinical variables correlated with 3,5-T_2_ concentrations and the effect of physiological and pathophysiological interventions. It would also be crucial to determine the effective concentrations achieved in experimental animals after the administration of exogenous 3,5-T_2_, and to compare them with the endogenous levels.

In the past, different immunoassays have provided variable results. Recently, a novel immunoassay has been developed and used in a large clinical series (7, 13, 14), but endogenous levels appear to be below the lower detection limit (200 nmol/L) in about one third of the subjects. In addition, the experience acquired with thyroid hormones assays suggests that immunological methods cannot be easily employed to determine tissue concentrations.

Mass spectrometry is a very promising analytical technique, since it displays significant advantages in terms of accuracy, sensitivity and specificity (15–17) and it has been successfully applied to tissue homogenates (18). So far, only a few papers reported 3,5-T_2_ measurements in humans or in animal models by mass spectrometry-based techniques.

Wang et al. (8) developed a LC-MS-MS method for the simultaneous measurement of 3,5-T_2_, 3,3′-T_2_, T_3_, rT_3_, and T_4_ in bovine serum samples and human serum from Standard Reference Materials. Both in bovine and in human serum the concentration of 3,5-T_2_ and 3,3′-T_2_ was below the lower detection limit of the method, namely 740 pg/mL (1.41 nmol/L) for 3,5-T_2_ and 920 pg/mL (1.75 nmol/L) for 3,3′-T_2_.

In 2014 Jonklaas et al. (19) published the results of a pilot study in which 3,3′T_2_ concentrations were evaluated by LC-MS-MS in 100 patients, with the primary aim to correlate T_2_ and clinical variables. Mean detected 3,3′-T_2_ levels were around 0.013–0.040 pmol/L and they could demonstrate an association between poor clinical conditions and low levels of T_2_ (19).

In a subsequent investigation, Hansen et al. (20) succeeded in detecting T_2_ in adult frog (*Xenopus laevis*) plasma and tadpole (*Rana catesbeiana*) serum. They developed a new analytical method by optimizing solid phase extraction, eventually choosing hydrophilic-lipophilic balanced mixed-mode polymeric materials (Plexa) and C18-materials to measure 11 thyroid hormone metabolites, including 3,5-T_2_ and 3,3′-T_2_, in 50 μl of serum or plasma, with a lower detection limit of 250 pg/mL (476 pmol/L). 3,5-T_2_ and 3,3′-T_2_ were detected in 80 and 30% of the samples, respectively, and their concentrations averaged 660 and 640 pmol/L, respectively.

Quite recently, Min Li et al. (21) optimized an LC-Q-TOF-MS method to determine thyroid hormone metabolites in placenta tissue. With this method, it was possible for the first time to track the presence of T_2_ (either 3,3′-T_2_ and 3,5-T_2_) in human and mice placenta at the concentration of 160–260 and 70–130 pg/g, respectively.

The method we developed has the advantage of a lower detection limit, which allowed 3,5-T_2_ and 3,3′-T_2_ detection in all our samples. This improvement is probably due to the higher sample volume and to the lower matrix effect allowed by the extensive washing steps included in the pre-analytical procedure. While this is promising, several drawbacks should be acknowledged: the needed samples volume is still too high for routine clinical use; the method is time consuming; the pre-analytical steps are too complex to be implemented on-line with the MS equipment. In addition, the human factor may be crucial, since each operator needed a significant training period before becoming able to get reproducible results. For these reasons, we believe that further developments are necessary before starting large-scale clinical investigations. In particular, sample preparation should be improved to make it easier and increase its reproducibility, throughput and ruggedness. If this can be achieved, it will become possible to establish a proper age- and gender-related reference range, and to investigate the effects of the different thyroid states and/or thyroid hormone supplementation.

While the present investigation should be regarded as a feasibility study, it may help to get further insight into T_2_ biochemistry and biology. Our results confirm that previous reports based on mass spectrometry-based techniques may have missed endogenous T_2_ because of their lower sensitivity. On the other hand, immunological techniques, particularly the recent method developed by Lemphul et al. provided control values for 3,5-T_2_ that are about 3-fold higher than we observed. The simplest explanation of this difference is the hypothesis that the different techniques allow identification of different T_2_ fractions. In general, immunological techniques rely on long incubation times and limited sample processing. In the presence of a high affinity antibody the analyte is expected to dissociate from its endogenous binding sites, and a good estimate of its total amount is obtained. On the other hand, mass spectrometry requires extensive sample processing and protein precipitation, making it likely that protein-bound analyte is lost.

T_3_ and T_4_ are known to be largely bound to plasma proteins, with a free/total ratio lower than 1/100, and a good agreement is usually observed between the results of mass spectrometry-based assays and the total amounts detected by immunological methods (9). Extensive binding to plasma protein has also be reported for 3-iodothyronamine, whose assay is complicated by additional pitfalls, such as oxidation by serum enzymes and adduct formation, further accounting for a large discrepancy between mass spectrometry and immunological detection (22). At present, very little is known about T_2_ binding to plasma proteins and/or serum metabolism. Adding T_2_ to reconstituted systems including specific plasma proteins and comparing the results of immunological and mass spectrometry-based assays might help to clarify this issue.

Our investigation was not aimed at evaluating the relationships between T_2_ level and other clinical variables. We could simply test the association between different iodothyronines. Interestingly, while 3,5-T_2_ and 3,3′-T_2_ levels were significantly related, neither T_2_ isomer showed any significant correlation with T_3_ or T_4_. This is consistent with the observation reported by Lemphul et al. (7) and it supports the hypothesis that T_2_ production may be specifically regulated. Because of this association, it would be interesting to investigate whether 3,3′-T_2_ assay may be used in the clinical field as an indirect index of 3,5-T_2_ availability.

In conclusion, we developed a method to measure 3,5-T_2_ and 3,3′-T_2_ in human serum samples, that significantly reduced matrix effect and showed high sensitivity and specificity. The method is promising, and further developments aimed at reducing its technical complexity might eventually provide a novel diagnostic tool amenable to large-scale clinical use.

## Ethics Statement

Serum samples were obtained from 28 patients, including 8 healthy volunteers and 20 women undergoing endocrinological screening and found to be euthyroid. Serum was obtained by using the remaining part of samples obtained for independent clinical indications. All subjects gave informed consent and since no additional blood drawings were performed, ethical committee approval was not required.

## Author Contributions

LL, NN, GS, ES, and MB performed the experiments. EC and TS collected the human samples. FS and SG analyzed the data. RZ, LL, and AS wrote the manuscript draft.

### Conflict of Interest Statement

The authors declare that the research was conducted in the absence of any commercial or financial relationships that could be construed as a potential conflict of interest.
